# Validating a Measure of Intrinsic Capacity in the Dunedin Study, a Sample of Midlife Adults

**DOI:** 10.1093/geroni/igaf122.2412

**Published:** 2025-12-31

**Authors:** J Kathy Xie, Avshalom Caspi, Renate Houts, Terrie Moffitt

**Affiliations:** Duke University, Durham, North Carolina, United States; Duke University, Durham, North Carolina, United States; Duke University, Durham, North Carolina, United States; Duke University, Durham, North Carolina, United States

## Abstract

Intrinsic capacity is a World Health Organization-supported multidimensional indicator of healthy aging that focuses on functional capacity rather than morbidity and mortality. Intrinsic capacity captures the sum of all mental and physical capabilities of the individual and includes 5 domains: locomotor, cognitive, psychological, sensory, and vitality. Consistent with the geroscience view that we must target aging processes early to extend healthspan, there is a need to validate intrinsic capacity in younger adults. Currently, the youngest participants in validation studies are 45, and most are over 65. We modelled intrinsic capacity using data from the Dunedin Study, a population-representative longitudinal birth cohort (N = 1037, Age=45). Measures comprising the 5 intrinsic capacity domains were attained using direct clinical testing and self- and other-report. Preliminary results from confirmatory factor analysis show that a bifactor model with 1 general factor (intrinsic capacity) and 5 specific subfactors (locomotor, cognitive, psychological, sensory, vitality) fit the data well, χ 2 value = 410.06 (df = 123); Comparative Fit Index = 0.93; Tucker-Lewis index=0.91; Root-Mean-Square Error of Approximation = 0.05. Future results will relate these factors to functional outcomes, such as biological aging, Alzheimer’s disease risk, and life satisfaction.

